# Underwater Wireless Sensor Networks Performance Comparison Utilizing Telnet and Superframe

**DOI:** 10.3390/s23104844

**Published:** 2023-05-17

**Authors:** Kaveripakam Sathish, Ravikumar CV, Mohd Nadhir Ab Wahab, Rajesh Anbazhagan, Giovanni Pau, Muhammad Firdaus Akbar

**Affiliations:** 1 School of Electronics Engineering, Vellore Institute of Technology, Vellore 632014, India; 2 School of Computer Sciences, Universiti Sains Malaysia, Minden 11800, Malaysia; 3 School of Electrical and Electronics Engineering, SASTRA University, Thanjavur 613401, India; 4 Faculty of Engineering and Architecture, Kore University of Enna, 94100 Enna, Italy; 5 School of Electrical and Electronics Engineering, Universiti Sains Malaysia, Eng. Campus, Nibong Tebal 14300, Malaysia

**Keywords:** CB-UWSN, energy, telnet, routing protocols, UWSN, superframe

## Abstract

Underwater Wireless Sensor Networks (UWSNs) have recently established themselves as an extremely interesting area of research thanks to the mysterious qualities of the ocean. The UWSN consists of sensor nodes and vehicles working to collect data and complete tasks. The battery capacity of sensor nodes is quite limited, which means that the UWSN network needs to be as efficient as it can possibly be. It is difficult to connect with or update a communication that is taking place underwater due to the high latency in propagation, the dynamic nature of the network, and the likelihood of introducing errors. This makes it difficult to communicate with or update a communication. Cluster-based underwater wireless sensor networks (CB-UWSNs) are proposed in this article. These networks would be deployed via Superframe and Telnet applications. In addition, routing protocols, such as Ad hoc On-demand Distance Vector (AODV), Fisheye State Routing (FSR), Location-Aided Routing 1 (LAR1), Optimized Link State Routing Protocol (OLSR), and Source Tree Adaptive Routing—Least Overhead Routing Approach (STAR-LORA), were evaluated based on the criteria of their energy consumption in a range of various modes of operation with QualNet Simulator using Telnet and Superframe applications. STAR-LORA surpasses the AODV, LAR1, OLSR, and FSR routing protocols in the evaluation report’s simulations, with a Receive Energy of 0.1 mWh in a Telnet deployment and 0.021 mWh in a Superframe deployment. The Telnet and Superframe deployments consume 0.05 mWh transmit power, but the Superframe deployment only needs 0.009 mWh. As a result, the simulation results show that the STAR-LORA routing protocol outperforms the alternatives.

## 1. Introduction

The ocean significantly impacts human life because it covers a third of the earth’s surface. Because of the rough nature of the undersea environment, only a tiny portion of the sea’s influence on the environmental state has been studied. Because of the discovery of a chemical poison, an aquatic natural resource, and oil spillage in recent years, monitoring has become increasingly crucial [1]. Underwater sensor nodes construct a small-scale Cluster-Based Underwater Wireless Sensor Network (CB-UWSN) by gathering data via point-to-point communication. Sensor Nodes (SNs) are often attached to surveillance or Global Positioning System (GPS) systems, although they can also be permanently deployed on the water’s surface in UWSNs. These networks are inexpensive, have few limits on their functions, and are simple to deploy. Using a wireless sensor network, often known as a WSN, is a significant step toward unraveling the mystery of underwater settings [2].

**Figure 1:**
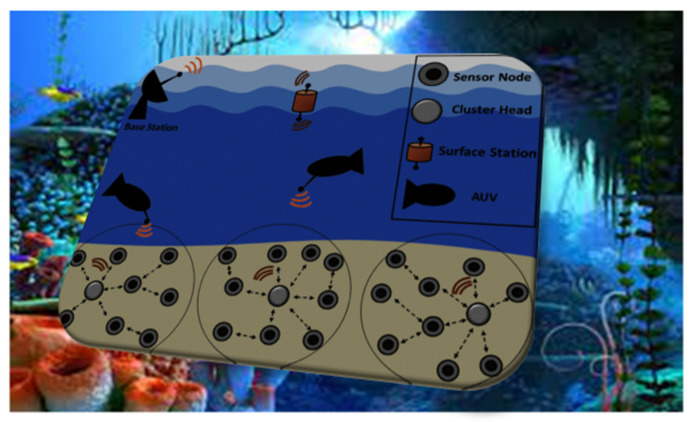
General architectural diagram of CB-UWSN.

The exploration of undiscovered oceans has inspired interest in the Internet of Underwater Things (IoUT), which intends to help solve problems in various industries, including the scientific community, security business, and others. The quantity of energy used and the quality of the networks used to transmit data are two significant issues in UWSN [3]. Because of the movement of the water, the function of SN is more complex and costly. Hop-to-hop communication consumes substantially less power than end-to-end transmission due to the periodic reorganization of the network’s structure [4,5]. When one node in the network wants to send data to another, the routing protocol establishes a path between the nodes in the proposed network. Between the source and the destination, routing table information is updated. Figure 1 depicts the general architectural diagram of CB-UWSN.

The routing protocol is a collection of rules used to find the most efficient way to transport data from its point of origin to its final destination. Network specs, channel parameters, and performance metrics vary, making it challenging to determine the optimal route [6]. Underwater wireless sensor networks, also known as UWSNs, are responsible for collecting data from their sensor nodes and relaying them to a central hub, which then processes the data after sharing them with other networks, such as the Internet. Single-hop communication is possible in minimal sensor networks because the base station and motes (sensor nodes) are so close to each other that they can communicate directly; however, in most UWSN applications, the coverage area is so large that thousands of nodes must be placed. In this case, multi-hop communication is required because the majority of sensor nodes are so far from the sink node (gateway) that they cannot communicate directly [7]. Direct communication is referred to as “one hop”, while indirect communication is called “many hops”. In multi-hop communication, sensor nodes produce and distribute their own content and act as a conduit for other sensor nodes to connect with the base station. In addition to producing and distributing their content, they manufacture and distribute their advertisements. Routing is an essential network layer function. Routing is choosing the most efficient path between a source and a destination node [8].

The purpose of this research is to compare the strengths and weaknesses of these two communication Telnet and Superframe protocols in the context of UWSNs. The goal of this analysis is to compare the two protocols’ abilities to maximize UWSN performance in key areas, such as energy efficiency, data transfer rate, and network dependability. The research goals include finding the best protocol for UWSN applications and dealing with challenges peculiar to underwater communication, such as high attenuation and limited bandwidth. The study’s specific goal is to answer the following questions:

1. How do the Telnet and Superframe protocols influence the power consumption of the UWSN?
2. How can reduced data transmission speeds affect the UWSN when using the Telnet and Superframe protocols?
3. How do Telnet and Superframe influence the dependability of the UWSN in comparison to other protocols?

This research attempts to provide answers to these questions in order to shed light on the strengths and drawbacks of the Telnet and Superframe protocols as they relate to UWSNs. This information can be used to improve underwater sensing and monitoring systems by informing the development of more effective and reliable UWSN communication protocols. Key contributions made by this manuscript are as follows:

➢ To evaluate the performance metrics of AODV, LAR1, OLSR, Fisheye, and STAR-LORA routing protocols in terms of energy in different modes, such as transmit, idle, and receive modes;
➢ To evaluate the energy efficiency of all the routing protocols as the number of underwater wireless sensor nodes increases;
➢ To evaluate the energy trade-off between receiver and transmitter modes;
➢ To propose a suitable routing protocol for an underwater wireless sensor network, taking into account the desired levels of transmitted and received power.

The remainder of this manuscript is organized as follows. Section 2 describes the related works. Section 3 briefly describes the background on communication protocols. Section 4 briefly discusses the proposed underwater network topology. Section 5 introduces the proposed CB-UWSN design parameters. Section 6 shows the obtained results and describes the discussion. Section 7 provides an overview of the entire manuscript and also the future directions.

## 2. Related Works

This section of the study examines prior research from the standpoint of network architecture, as well as the many performance indicators that support the concept of extending network life.

Yildiz et al. [9] investigate strategies to optimize the number of packets sent in order to extend the life of UWSNs. This is done in order to determine how to make the most of UWSNs and maximize their potential benefits. To guarantee that a network lasts as long as feasible, it is critical to consider not only the time it takes for a packet to transmit, but also the overall size of the packet. Furthermore, in order to reduce the amount of power consumed by the network, we accurately simulate the connection layer.

Alkindi et al. [10] investigate a grid-based routing technique for UWSN as a means of addressing mobility concerns. The latency, energy usage, network density, and packet delivery ratio are all topics of discussion.

Bhattacharya et al. [11] utilize a universal wireless sensor network with a grid topology. In this study, both the efficiency of various network configurations and the utilization of various energy modalities are investigated.

Wang et al. [12] look at EAVARP, which is an energy-conscious and void-avoiding routing algorithm for wireless sensor networks. Transmission, vacancy, and flooding cycles have no effect on UWSN because it is immune to them.

Mohan et al. [13] investigate interference-free localization routing for ultra-wideband sensor networks, with the intention of reducing the energy hole. The study details the overall amount of energy used, the total number of dropped packets, the total number of dead nodes, and a packet that was received at the sink.

These studies, on the other hand, are more general inquiries into WSN energy use. The UWSN environment, on the other hand, is unique, and the underwater acoustic communication paradigm differs from that of typical WSNs. Furthermore, the aforementioned models are largely concerned with tackling general routing concerns in WSNs while ignoring clustering issues. This paper explores the optimization of energy consumption in clustered, routing-based underwater wireless sensor networks (UWSNs) and presents a CB-UWSN system for energy balancing to successfully balance the energy consumption of underwater wireless sensor nodes. This paper also tackles the optimization of energy consumption in UWSNs via clustered routing.

## 3. Background on Communication Protocols

Underwater communication protocols are specialized sets of rules and procedures that are designed to facilitate the transmission and reception of data between underwater devices, such as sensors, vehicles, and buoys. These protocols are necessary because the underwater environment poses unique challenges that are not present in other communication scenarios. One major challenge of underwater communication is the high attenuation of electromagnetic waves in water, which limits the range and bandwidth of wireless communication protocols. As a result, most underwater communication protocols use acoustic signals to transmit data. Acoustic signals have a longer range in water than electromagnetic waves and can travel through the water with lower attenuation [14].

There are various types of underwater communication protocols, each with their own advantages and limitations. For example, some protocols, such as acoustic modems, are designed for high data rate applications and can transmit data at rates of several megabits per second over short distances. Other protocols, such as acoustic telemetry, are designed for long-range communication and can transmit data over distances of several kilometers, but at lower data rates. Underwater communication protocols also differ in terms of their operating frequency, modulation scheme, and error correction techniques. These parameters are important for optimizing the communication link and ensuring reliable data transfer between devices [15].

### 3.1. Ad Hoc On-Demand Distance Vector (AODV)

The only thing that needs to be altered to implement AODV while employing a reactive traffic routing protocol is the routing of existing paths. A node’s routing tables are where routing information is stored. Each mobile node’s “next-hop routing database” stores information about the mobile nodes to which that mobile node is capable of connecting in the next hop. If an entry in a routing table has not been used in a particular amount of time, it can be removed from the table. The destination sequence number is used on demand by both the AODV and the DSDV, but the AODV more frequently. If the transmitting node is unable to determine whether or not there is a path to the destination, AODV will begin looking for one [16].

### 3.2. Fisheye State Routing (FSR)

A multilayer and table-driven routing method for use in ad hoc networks, Fisheye State Routing is also known by its acronym FSR. This method utilizes the scope approach. The amount of overhead that is associated with routing in highly large networks that are prone to rapid change and dynamic behavior is something that needs to be reduced. In accordance with the methodology that underpins its scope, the connection state changes are periodically broadcast at a predetermined frequency. The entirety of the network is segmented into a variety of scopes, each of which is determined by the number of hops that are required to reach a particular node. These hops are counted from the root node backwards. Nodes that are considered to be within this distance of one another are referred to as inner nodes, while nodes that are further apart are referred to as outer nodes.

The transmissions of the most recent connection state updates are made available to neighbors across a wide range of frequencies. Information is delivered at a lower frequency, while simultaneously increasing its frequency while connecting with neighbors that are closer to the sender. The accuracy of the connection state updates that the nodes receive as a direct result of this is much improved. It improves the precision of the path that packets are sent across when they are transferred [17].

### 3.3. Location-Aided Routing 1 (LAR1)

In wireless ad hoc networks, the LAR protocol is deployed as a kind of location-based and reactive routing. It accomplishes this by utilizing three separate packets, namely, the route request, the route reply, and the route error, in order to send and receive information between nodes and maintain stable connections. The LAR on-demand routing protocol functions in a manner that is comparable to that of the DSR (dynamic source routing) protocol. The LAR protocol, as opposed to the DSR protocol, makes use of location data in order to establish the borders of the “request zone”, which is the area in which it is feasible to discover new routes. This zone is defined in contrast to the DSR protocol, which does not make use of location data. As a direct result of this, the route requests will not be broadcast to the full of the network, but rather will only be transmitted by the nodes that are a part of the request zone. The originator provides a forecast about a spherical region that is termed the predicted zone. This region is the location in which the target object is most likely to be discovered at this same current time. The prediction is made by using data from the past [18].

### 3.4. Optimized Link State Routing Protocol (OLSR)

OLSR (optimized link state routing) is a prominent type of dynamic routing technique. Frequent transmissions of routing information from each network node ensure that all network nodes have the same comprehensive perspective. The protocol incurs significant extra expense due to its periodic structure. The problem is resolved since OLSR limits the amount of traffic that can be transferred. To achieve this purpose, multi-point relays (MPRs) are used; these devices are in charge of relaying and routing messages. Each node selects MPRs from among its neighboring nodes. It is advised that for optimal performance, a node choose MPRs that allow it to connect with at least one neighbor that is reachable over a hop-and-a-half path. MPRs are in charge of relaying control traffic generated by other nodes [19].

### 3.5. Source Tree Adaptive Routing—Least Overhead Routing Approach (STAR-LORA)

The STAR protocol was created to be used by both mobile and stationary nodes in a network context, such as the Internet or ad hoc networks. As a result, the protocol can be employed in any situation. The surrounding routers of a STAR router will require access to the source routing tree’s settings while configuring it. The source routing tree stores every possible connection that the router needs to communicate with any host or combination of hosts anywhere on the Internet or in the ad hoc network. A router will only change the source routing tree it uses to direct traffic after learning about new destinations, the risk of loops, node failure, or network breakup. This reduces the router’s power consumption and increases its data transfer capability. The STAR routing protocol can be implemented in a variety of ways, including using an optimum routing algorithm (ORA) or a least-overhead routing strategy (LORA) [20].

Telnet (Telecommunication Network) is an abbreviation for a network protocol that can be utilized across both Internet and LAN connections. This network protocol enables bidirectional, interactive communication over wide-area networks, such as the Internet, as well as more intimate networks, such as local area networks (LANs), and interactive, two-way communications, such as instant messaging. In most cases, Telnet can be used to establish a virtual terminal connection to the command line interface of a remote computer. This link, which is a byte-oriented data connection with 8 bits, is supported by the transmission control protocol (TCP). In the same frequency channel, dispersed user data are provided with Telnet control information. Because the user’s PC initiates contact, it is referred to as the “local computer” [21].

The term “remote computer” refers to the machine at the other end of a connection. The remote computer could be in the next room, in another city, or even in another country. Telnet is a protocol that allows a user to log in from any computer on the network. A remote session can be started by simply selecting a host machine. Everything typed after that gets forwarded to the other computer and vice versa until the session terminates.

Telnet is an application that, when run, connects the user’s computer to a server located somewhere on the network. After that, we may use the Telnet application to input commands, which will be performed just as if they were entered on the server’s console. This allows one to control the server and connect with other network server operators. Before beginning a Telnet session, you must first log in to the server using a valid username and password. Remote control of Web servers via the Telnet protocol is typical practice [22].

**Figure 2:**
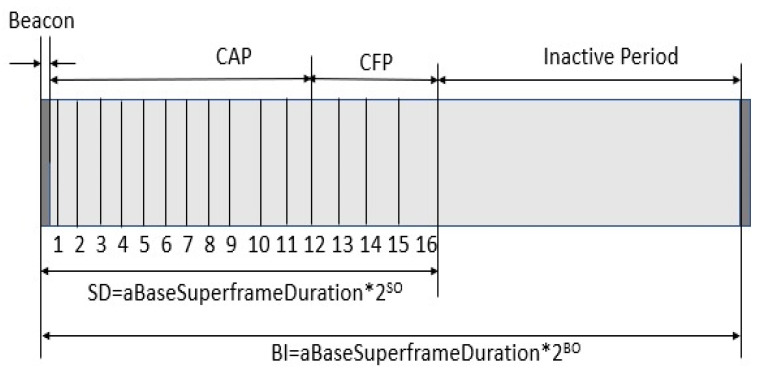
Superframe design based on IEEE 802.15.4.

Figure 2 Depicts a Superframe design based on IEEE 802.15.4. The PAN coordinator can purposely limit the amount of time that can be spent on a channel by using a Superframe structure, as shown in Figure 1. The structure of the Superframe is created by the coordinator’s beacon, and it is then divided into sixteen slots of the same size, as seen in Figure 1. The beacon interval can range from 15 milliseconds to 245 s, and the frame is broadcast in the very first slot of the Superframe.

Beacons are used to identify a PAN, synchronize the devices associated with the PAN, and explain the Superframe’s architecture. The time interval between beacon frames is divided into 16 equal parts, regardless of the time interval between Superframes. Any moment in time throughout the time slot may be used for data transmission; however, all data must be sent or received before the next Superframe period begins. The beacon frame contains, in addition to the Superframe specification and the current node notification, the pending node message and the current node notification.

There is a distinction to be made between the Superframe’s active period and its inactive phase. During moments of inactivity, the device will automatically transition to a lower-power mode. There are two unique time frames within the active period, known as the contention access period (CAP) and the contention-free period (CFP). Devices attempting to send data frames in the CAP use a procedure known as slotted CSMA/CA to acquire access to the wireless channel. The CFP, on the other hand, is used to transport data frames with assured quality of service since it is split into guaranteed time slots (GTSs). A coordinator, for example, might provide a GTS, particularly for applications that require real-time updates or a large amount of bandwidth.

Moore’s research on the emission of electric dipoles at the boundary surface dividing two media aroused interest in the use of electromagnetic waves to transport information between antennas immersed in a conducting medium. Moore and Blair investigated ELF transmissions between antennas submerged in a conducting half-space medium. The study was based on the assumption that a lateral wave with three components is the primary mode of communication between submerged antennas. These components are as follows: (1) a wave that travels along the sea surface; (2) a wave that travels directly from the transmitting dipole to the water’s surface; and (3) a wave that travels perpendicular to the water’s surface from the water’s surface to the receiving dipole.

**Figure 3:**
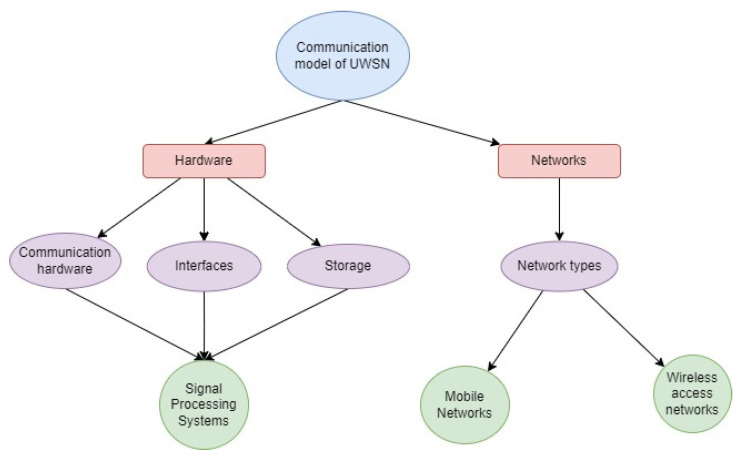
Communication model of UWSN.

With this notion, direct path attenuation was projected to be substantially greater than lateral wave attenuation. Waves reflected from the ocean floor were not taken into account because they would result in significantly more attenuation than the straight path. While very low frequency (ELF) transmission was considered, the displacement current commonly associated with a medium losing conductivity was disregarded. Figure 3 represents the communication model of the UWSN with various hardware and network assemblies.

## 4. Proposed Underwater Network Topology

**Figure 4:**
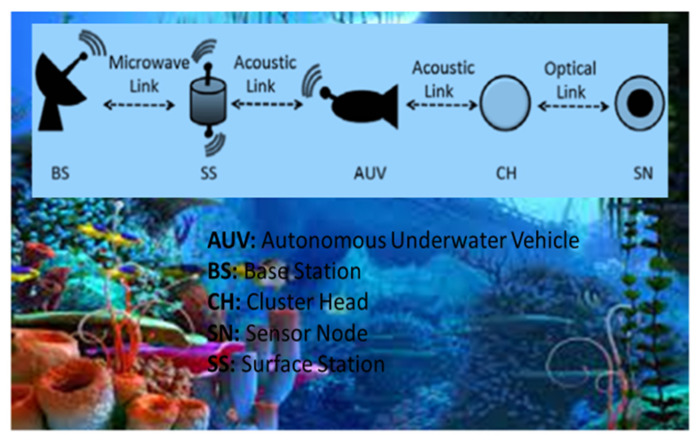
Internal link communication of CB-UWSN.

Existing networks are accessible with CBR as a deployment application. Telnet and Superframe applications are considered in the proposed network, and the settings for Telnet and Superframe applications are then compared. In the QualNet Simulator, the proposed scenario features a 1500-by-1500 square meter design with 250 nodes connecting the Telnet and Superframe programs, 75 of which are sensor devices, 25 are ship devices, and 150 are node devices. The simulation lasts a total of five hundred seconds. The node mobility model used is Random Waypoint Mobility, with a minimum speed of 1.5 m per second and a maximum speed of 2 to 11 m per second [23,24]. LAR1, OLSR, Fisheye, STAR-LORA, and AODV are used as the first routing protocol. Figure 4 shows the internal link communication of CB-UWSN, where it has AUV: Autonomous Underwater Vehicle, BS: Base Station, CH: Cluster Head, SN: Sensor Node, and SS: Surface Station, with the communication links Microwave link, Acoustic link, and Optical link to establish the connection between the base station to the sensor nodes via the surface station.

**Figure 5:**
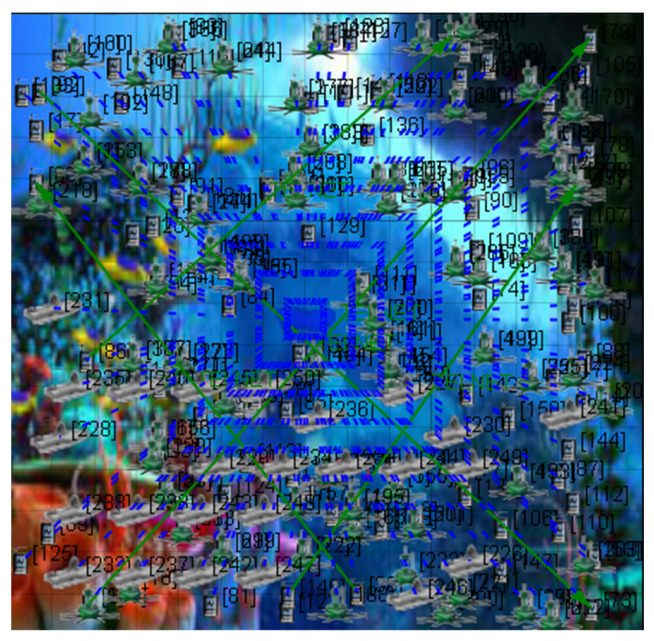
Network scenario with CB-UWSN in XY Visualization.

**Figure 6:**
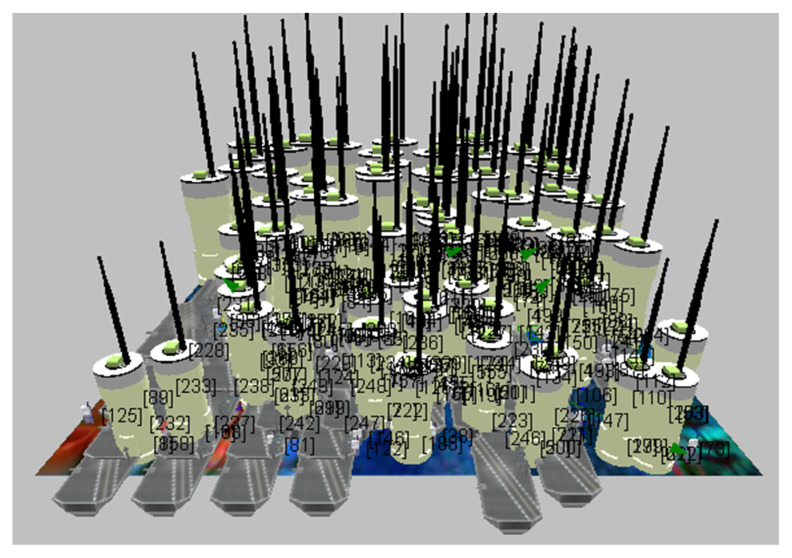
Network scenario with CB-UWSN in 3D Visualization.

**Figure 7:**
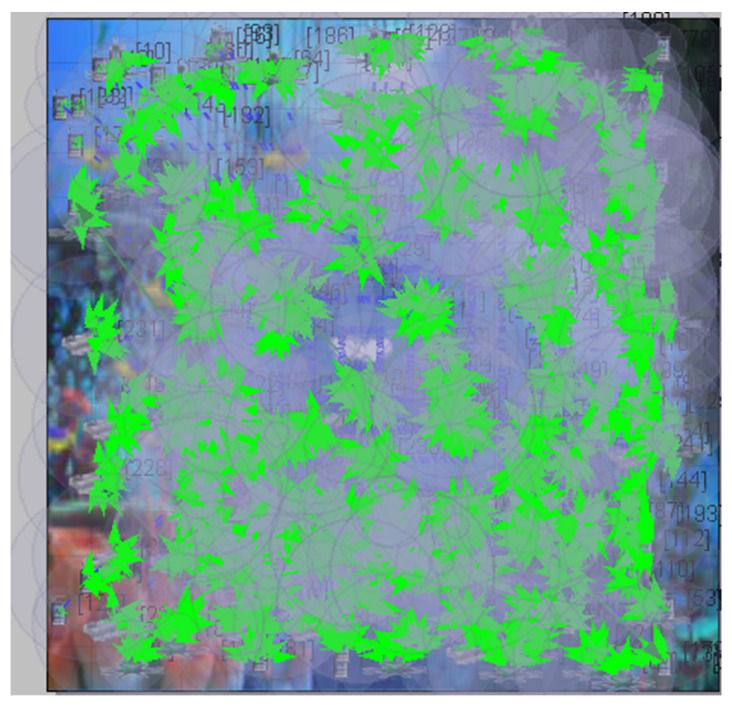
Runtime view of Network scenario with CB-UWSN in XY Visualization by deploying Telnet.

**Figure 8:**
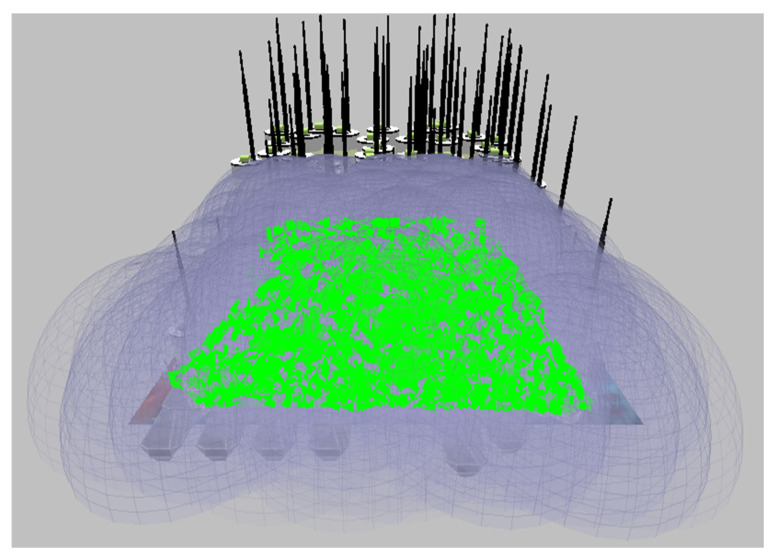
Runtime view of Network scenario with CB-UWSN in 3D Visualization by deploying Superframe.

The graphs in the simulator were considered after the test was completed. Thus, the required performance metric, energy utilized in the transmit and receive modes, is obtained. Figure 5 and Figure 6 show the proposed CB-UWSN underwater wireless communication scenario with multiple nodes in X-Y and 3D visualization for 250 nodes. Figure 7 and Figure 8 show the runtime proposed scenario for CB-UWSN underwater wireless communication with various nodes in both X-Y and 3D visualization for 250 nodes. The purpose of Algorithm 1 is to cluster the sensor nodes in an underwater wireless sensor network based on their residual energy. The input to the algorithm is the number of sensor nodes k and the set of sensor nodes (n_1_, n_2_, n_3_, …, n_k_). The output of the algorithm is the clustering of the sensor nodes.
**Algorithm 1:** Identifying clusters for UWSNInput: The number of sensor nodes k, (n_1_, n_2_, n_3_, …, n_k_) Output: Clustering of sensor node       Step 1: Placement of k number of sensor nodes (n_1_, n_2_, n_3_, …, n_k_)       Step 2: Find the energy between the sensor nodes by using RE_k_ = IE_k_ − CE_k_
      Step 3: Founded upon the high residual energy of node is the cluster              Where RE_k_ = Residual Energy of kth node              IE_k_ = Ideal Energy of kth node             CE_k_ = Consumed Energy of kth node

The algorithm consists of three steps:

**Step 1:** Placement of k number of sensor nodes: this step involves placing the k sensor nodes in the underwater environment.

**Step 2:** Find the energy between the sensor nodes: this step involves finding the energy between the sensor nodes by using the formula RE_k_ = IE_k_ − CE_k_, where RE_k_ is the residual energy of the kth node, IE_k_ is the ideal energy of the kth node, and CE_k_ is the consumed energy of the kth node.

**Step 3:** Founded upon the high residual energy of the node is the cluster: This step involves clustering the sensor nodes based on their residual energy. The sensor node with high residual energy is selected as the cluster head, and the remaining nodes are assigned to the cluster head.

Overall, the algorithm aims to form clusters of sensor nodes based on their residual energy, which can help in prolonging the network lifetime and improving the network performance.

To simplify the process of building and evaluating the propagation model more easily, we assume that the 2 insulated magnetic dipoles are co-planar (=90 degrees) and parallel to the surface of the water. Furthermore, we assume that the propagation medium is homogeneous and isotropic. Despite the fact that dielectric phenomena can explain large signal losses at greater distances, and a lossy conducting medium can explain significant signal losses near the transmitter, we consider the theoretical foundation, which includes both the displacement current and the conduction current. If we assume that the received signal has a uniform distribution and acts like a plane wave, we may compute the average power density Equations (1)–(3).

Equations (1)–(3) describe the behavior of electromagnetic waves in an underwater environment. These equations are important in the design and analysis of underwater communication systems, where it is crucial to understand the propagation of electromagnetic waves in this challenging environment.

(1) $$\left|E_{\emptyset }\right|=\frac{\mu \omega IdA}{4\pi r^{2}}exp(-\alpha r)\sqrt{{\left(\beta r\right)}^{2}+{\left(1+\alpha r\right)}^{2}}sin\theta$$

Equation (1) represents the electric field strength at a distance r from the source. It depends on the parameters μ, ω, Id, A, α, β, and θ.

Where

μ is the magnetic permeability of the medium;
ω is the angular frequency of the signal;
Id is the current density of the source;
A is the area of the source;
α and β are attenuation constants that depend on the properties of the medium;
θ is the angle between the direction of the electric field and the normal direction to the surface.

(2) $$S=\frac{{\left|E_{\emptyset }\right|}^{2}}{2}exp(-2\propto r)Re\left[\frac{1}{\eta_{c}^{*}}\right]$$

Equation (2) calculates the signal power density S at a distance r from the source. It depends on the electric field strength $E_{\emptyset }$, the attenuation constant α, and the intrinsic impedance $\eta_{c}$ of the medium.

(3) $$\eta_{c}=\sqrt{\frac{j\omega \mu }{\sigma +j\omega \varepsilon }}$$

Equation (3) defines the intrinsic impedance $\eta_{c}$, which depends on the properties of the medium: the magnetic permeability μ, the conductivity σ, and the dielectric constant ε. The following algorithms represent the selection of the cluster head node (CHN) and energy dissipated by the cluster head and the network parameters are updated as in Table 1.

Algorithm 2 is used for selecting the cluster head node (CHN) in the CB-UWSN. In this algorithm, each sensor node in the network is assigned a pair of values, l_k_ and h_k_, which represent the lower and higher thresholds, respectively. The algorithm uses a random number generator to determine whether a node should be selected as the CHN or a normal node based on these threshold values. The algorithm works as follows.
**Algorithm 2:** Selection cluster head node (CHN) for UWSN if node = CHN then       if rand ≤ l_k_ then       sensor node = CHN else sensor node = normal node end else       if rand ≤ h_k_ then        sensor node = CHN       else        sensor node = normal node        end end       Where l_k_ = lower value of kth node               hk = higher value of kth node

If the current node is already the CHN, the algorithm checks if a randomly generated number is less than or equal to the lower threshold value l_k_. If it is, the current node remains the CHN; otherwise, the current node becomes a normal node. If the current node is not the CHN, the algorithm checks if a randomly generated number is less than or equal to the higher threshold value h_k_. If it is, the current node becomes the CHN; otherwise, the current node remains a normal node. By using this algorithm, the network can dynamically select the CHN based on the current values of l_k_ and h_k_ for each sensor node. This can help to balance the energy consumption and extend the lifetime of the network. Algorithm 3 is used to calculate the energy dissipated by the cluster head in a UWSN. The algorithm consists of five steps.
**Algorithm 3:** Energy dissipated by the cluster head      Begin      Step 1: Network Initialization      Step 2: If dmin > E_FS_ then the energy of kth node      Step 3: Energy of kth node (E_k_) = (RE_max_ − ReE_min_)/(ReE_max_ − RE_min_)      Step 4: If dmin ≤ E_FS_ then the energy of kth node      Step 5: Energy of kth node (E_k_) = (ReE_min_ − RE_max_)/(RE_min_ − ReE_max_)      End      Where      d_min_ = minimum distance between the sensor nodes      E_FS_ = Energy of free space      E_k_ = Energy of kth node      RE_max_ = maximum residual energy      RE_min_ = minimum residual energy      ReE_max_ = maximum remaining energy      ReE_min_ = minimum remaining energy

**Step 1:** Network Initialization. This step is used to initialize the network before calculating the energy dissipated by the cluster head.

**Step 2:** If d_min_ > E_FS_ then the energy of kth node. In this step, if the minimum distance between the sensor nodes (d_min_) is greater than the energy of free space (E_FS_), the energy of the kth node is calculated.

**Step 3:** Energy of kth node (E_k_) = (RE_ma_x − ReE_min_)/(ReE_max_ − RE_min_). The energy of the kth node (E_k_) is calculated using the maximum residual energy (RE_max_), minimum residual energy (RE_min_), maximum remaining energy (ReE_max_), and minimum remaining energy (ReE_min_). The formula used is (RE_max_ − ReE_min_)/(ReE_max_ − RE_min_).

**Step 4:** If d_min_ ≤ E_FS_ then the energy of kth node. In this step, if the minimum distance between the sensor nodes (d_min_) is less than or equal to the energy of free space (E_FS_), the energy of the kth node is calculated.

**Step 5:** Energy of kth node (E_k_) = (ReE_min_ − RE_max_)/(RE_min_ − ReE_max_). The energy of the kth node (E_k_) is calculated using the maximum residual energy (RE_max_), minimum residual energy (RE_min_), maximum remaining energy (ReE_max_), and minimum remaining energy (ReE_min_). The formula used is (ReE_min_ − RE_max_)/ (RE_min_ − ReE_max_).

Overall, the algorithm is used to calculate the energy of the cluster head in a UWSN based on the distances between the sensor nodes and the energy of free space.

In underwater environments, the energy of free space (EFS) value is generally lower than in air due to the attenuation of radio waves caused by the high absorption and scattering of electromagnetic waves in water. The actual value of EFS in underwater environments can vary, depending on such factors as the frequency of the radio signal, the salinity and temperature of the water, and the presence of obstacles or reflections. Generally, EFS values in underwater environments range from tens to hundreds of microjoules per bit per meter (μJ/bit/m). In our simulation, we have considered the E_FS_ as 5 (μJ/bit/m).

The value of d_min_ in Algorithm 2 refers to the minimum distance between the sensor nodes in a cluster. This value is typically chosen based on the specific requirements of the application and the characteristics of the underwater environment, such as the maximum communication range of the sensor nodes, the desired sensing resolution, and the level of interference in the environment. The value of d_min_ is an important parameter in determining the size and shape of the clusters in the CB-UWSN, which will vary from 1 m to 3 m, based on the cluster node distance. It ensures that the sensor nodes within a cluster are in close proximity to one another, which can improve communication efficiency and reduce energy consumption.

## 5. Proposed CB-UWSN Design Parameters

When a sensor node is part of a wireless sensor network, the three operations that consume the most energy are data collecting, processing, and transmission. The capture energy is lost while the sample is being taken, the signal is being processed, the analog-to-digital conversion is taking place, and the capture probe is being engaged. The entire processing energy is composed of two components: switching power and leakage power. The software will toggle between the supply voltage and the total switched capacitance to determine the switching energy (by executing a software). However, even when the processing unit is not actively working, it consumes energy, which is known as leakage energy. Power in communication is divided into two components: reception energy, which is necessary for data receipt, and transmission energy (required for data transmission). Several elements are considered while estimating the required amount of power, including the data load, the distance that must be transferred, and the radio module parameters. One of the properties that define a signal is its strength while it is being conveyed. If the transmission strength is high, the signal will be able to go further, but at the penalty of using more energy. The amount of energy necessary to acquire and process data is often relatively low and can be regarded as insignificant when compared to the amount of energy required for real communication. Given that communication accounts for the great majority of a sensor node’s overall energy consumption, we shall concentrate entirely on this element.

**Consumption of Transmit Mode Energy:** It provides information regarding the amount of power that is consumed by a network throughout the process of transmitting data from one node to another [25].

**Consumption of Receive Mode Energy:** It provides information regarding the amount of power that is consumed by a network throughout the process of receiving data from one node to another [26].

**Consumption of Idle Mode Energy:** Using up lots of energy when in idle mode, it provides information on the amount of power that is used by a network while it is not sending or receiving data from one node to another. This occurs when the network is not actively transmitting or receiving data [27].

Energy consumption is calculated using Equation (4).

(4) $$EnergyConsumption=\frac{E_{total}}{N\times Pt_{S}\times 50}$$

where Pt_s_ is the successful packet reception, N is the number of nodes, and E_total_ is the total energy consumption

Traditionally, these UWSNs require a lot of power to function. However, lowering the system’s power requirements extends the lifetime of the sensor devices and frees up space for battery-powered applications [28,29,30]. Battery-powered gadgets enable a wide range of use cases and provide options for low-return applications. Low-power wireless sensor networks come into play here [31,32]. The term “low power” refers to wireless sensor networks built to minimize the power needs of individual wireless sensor nodes, which is the key to increasing the lifetime of WSNs. In order to reduce overall power consumption, low-power wireless sensor networks regulate the “awake time” of the devices (such as mobile) and limit the current drawn during their “sleeping” states [33,34]. To do this, these networks adjust the power settings of the connected devices to states such as “always on”, “standby”, and “hibernation” [35].

## 6. Results and Discussion

**Figure 9:**
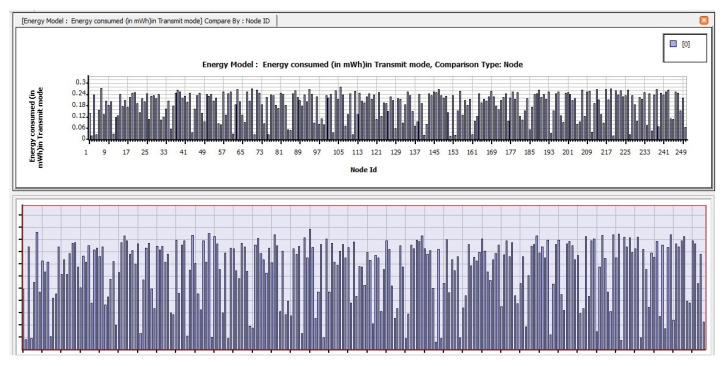
Consumption of energy when operating in the Transmit mode of STAR-LORA for Telnet deployment.

**Figure 10:**
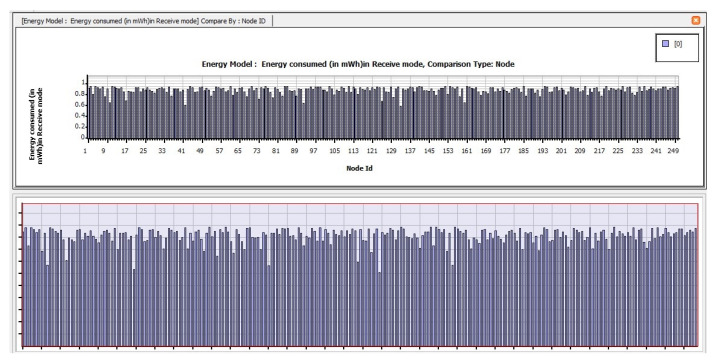
Consumption of energy when operating in the Receive mode of STAR-LORA for Telnet deployment.

**Figure 11:**
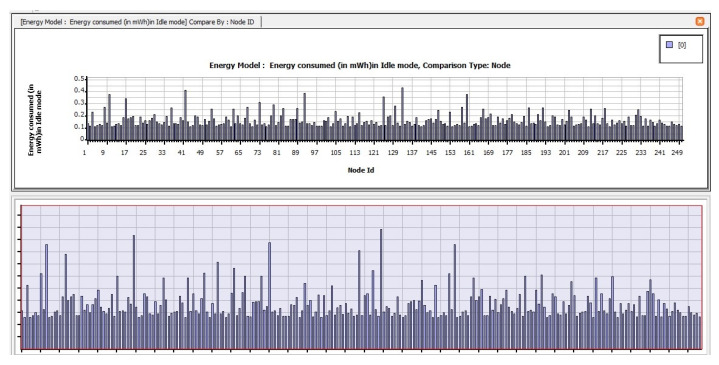
Consumption of energy when operating in the Idle mode of STAR-LORA for Telnet deployment.

The following are the findings of assessing the planned CB-UWSN network’s performance characteristics in the Telnet and Superframe applications for deploying 250 nodes. Figure 9 depicts the amount of energy consumed by transmit mode routing protocols, such as STAR-LORA, with the applications of Telnet for 250 nodes. Figure 10 depicts the amount of energy consumed by receive mode routing protocols, such as STAR-LORA, with the applications of Telnet for 250 nodes. Figure 11 depicts the amount of energy consumed by idle mode routing protocols, such as STAR-LORA, with the applications of Telnet for 250 nodes.

**Figure 12:**
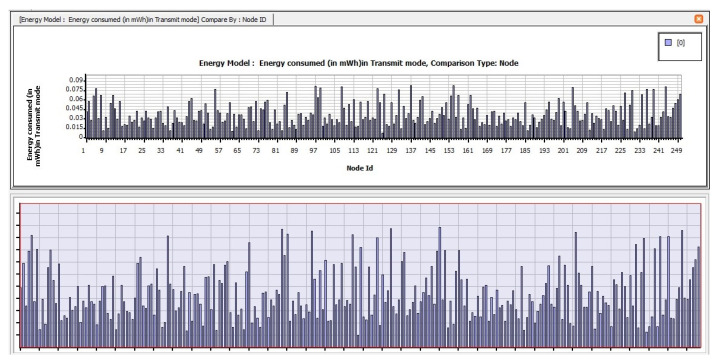
Consumption of energy when operating in the Transmit mode of STAR-LORA for Superframe deployment.

**Figure 13:**
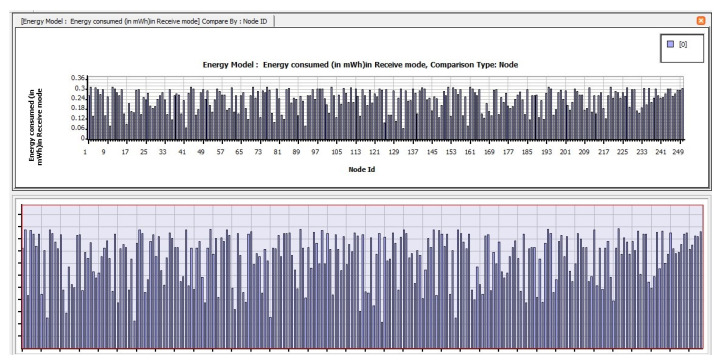
Consumption of energy when operating in the Receive mode of STAR-LORA for Superframe deployment.

**Figure 14:**
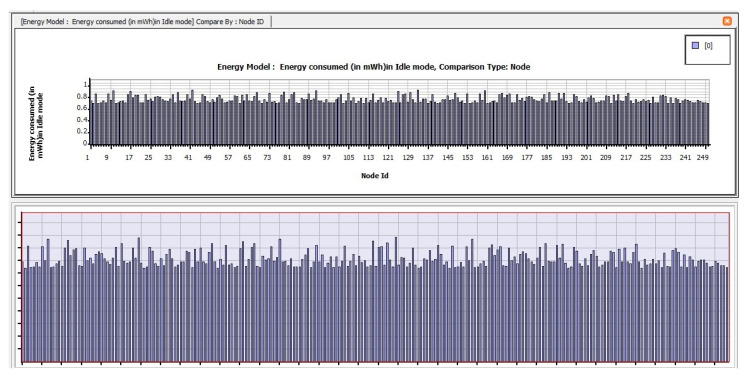
Consumption of energy when operating in the Idle mode of STAR-LORA for Superframe deployment.

Figure 12 depicts the amount of energy consumed by transmit mode routing protocols, such as STAR-LORA, with the applications of Superframe for 250 nodes. Figure 13 depicts the amount of energy consumed by receive mode routing protocols, such as STAR-LORA, with the applications of Superframe for 250 nodes. Figure 14 depicts the amount of energy consumed by idle mode routing protocols, such as STAR-LORA, with the applications of Superframe for 250 nodes.

The UWSN network has the following performance metrics for Telnet and Superframe applications, as shown in Table 2 for AODV, Table 3 for Fisheye, Table 4 for STAR-LORA, Table 5 for LAR1, and Table 6 for OLSR.

### 6.1. Consumption of Energy (mWh) in the Transmit Mode

**Figure 15:**
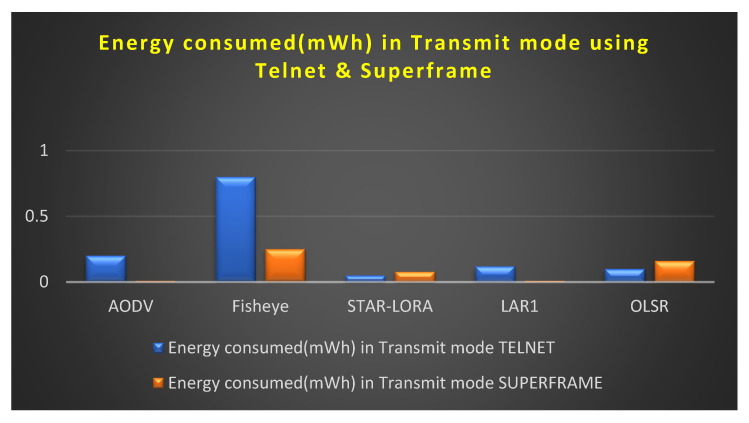
Consumption of energy when operating in the Transmit mode using Telnet and Superframe deployment.

Figure 15 depicts the amount of energy consumed by transmit mode routing protocols, such as AODV, LAR1, OLSR, Fisheye, and STAR-LORA, when combined with the applications of Telnet and Superframe for 250 nodes. As can be seen in Table 7, the minimum amount of transmitting energy that is necessary for the AODV, LAR1, OLSR, Fisheye, and STAR-LORA routing protocols to send data at their maximum size in the proposed CB UWSN is 0.009 mWh for LAR1 in the Superframe deployment application and 0.05 mWh for STAR-LORA in the Telnet deployment application. For STAR-LORA, the value for this minimum amount of transmit energy is the goal of the CB-UWSN, which is to reduce the amount of transmit power used as much as feasible. Regarding speed and dependability, no other routing protocol can compete with STAR- LORA in the Telnet deployment application and LAR1 in the Superframe deployment application.

### 6.2. Consumption of Energy (mWh) in the Receive Mode

**Figure 16:**
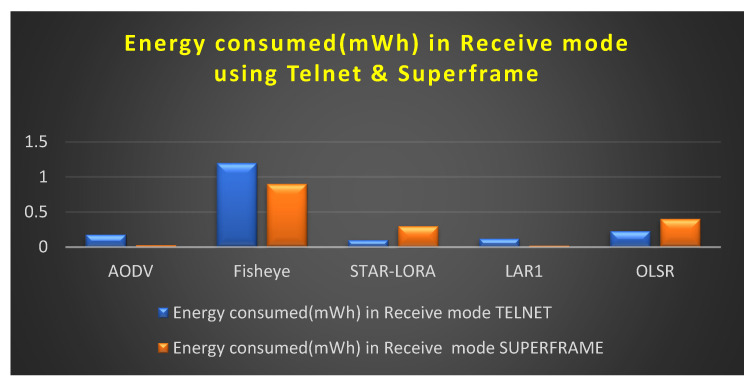
Consumption of energy when operating in the Receive mode using Telnet and Superframe deployment.

Figure 16 depicts the amount of energy consumed by transmit mode routing protocols, such as AODV, LAR1, OLSR, Fisheye, and STAR-LORA, when combined with the applications of Telnet and Superframe for 250 nodes. As can be seen in Table 7, the minimum amount of transmitting energy that is necessary for the AODV, LAR1, OLSR, Fisheye, and STAR-LORA routing protocols to send data at their maximum size in the proposed CB UWSN is 0.021 mWh for LAR1 in the Superframe deployment application and 0.1 mWh for STAR-LORA in the Telnet deployment application. For STAR-LORA, the value for this minimum amount of received energy is the goal of the CB-UWSN, which is to reduce the amount of transmit power used as much as feasible. Regarding speed and dependability, no other routing protocol can compete with STAR-LORA in the Telnet deployment application and LAR1 in the Superframe deployment application.

### 6.3. Consumption of Energy (mWh) in the Idle Mode

**Figure 17:**
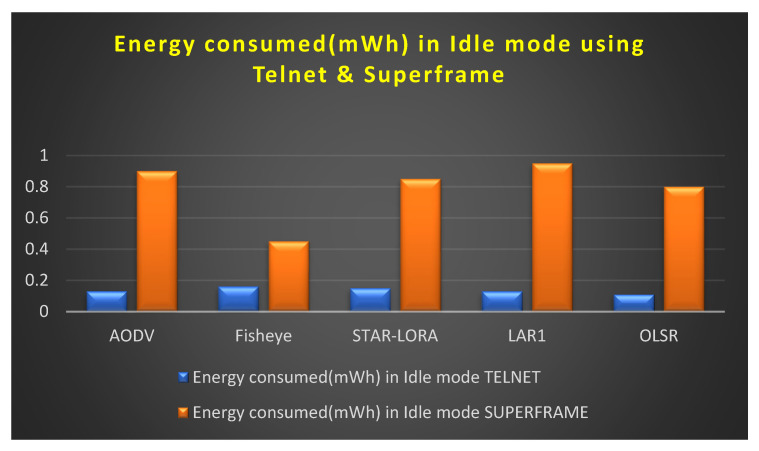
Consumption of energy when operating in the Idle mode using Telnet and Superframe deployment.

Figure 17 depicts the amount of energy consumed by transmitting mode routing protocols, such as AODV, LAR1, OLSR, Fisheye, and STAR-LORA, when combined with the applications of Telnet and Superframe for 250 nodes. As can be seen in Table 7, the minimum amount of transmitting energy that is necessary for the AODV, LAR1, OLSR, Fisheye, and STAR-LORA routing protocols to send data at their maximum size in the proposed CB-UWSN is 0.45 mWh for Fisheye in the Superframe deployment application and 0.11 mWh for OLSR in the Telnet deployment application.

Table 7 shows the comparison result values for all the routing protocols, such as AODV, LAR1, OLSR, Fisheye, and STAR-LORA, when combined with the applications of Telnet and Superframe for 250 nodes.

#### Research Questions and Answers

**Question 1: How do the Telnet and Superframe protocols influence the power consumption of the UWSN?**

**Answer 1:** The Telnet and Superframe protocols can have different effects on the power consumption of an underwater wireless sensor network. Telnet is a simple, lightweight protocol that is commonly used in networking applications to establish a remote terminal session with a device. Superframe, on the other hand, is a more complex protocol that is designed specifically for low-power wireless sensor networks, including UWSNs.

One way that Telnet and Superframe can influence power consumption is through their communication overhead. Telnet has a relatively low communication overhead, which means that it requires less power to transmit and receive data compared to Superframe. The same has been shown in our simulation results in the Telnet deployment application, the STAR-LORA routing protocol consumed 0.1 mWh of energy, whereas the LAR1 routing protocol consumed 0.021 mWh of energy in the Superframe deployment application. LAR1 used 0.009 mWh of energy in transmit mode when running the Superframe deployment application, whereas STAR-LORA used 0.05 mWh of energy while running the Telnet deployment application. This makes Telnet a good choice for applications where power consumption is a critical concern.

Another way that Telnet and Superframe can influence power consumption is through their data transmission rate. Telnet is a relatively slow protocol compared to Superframe, which can transmit data at higher rates. Higher data transmission rates can consume more power, but can also reduce the amount of time that devices need to be active, which can lead to overall energy savings.

**Question 2: How can reduced data transmission speeds affect the UWSN when using the Telnet and Superframe protocols?**

**Answer 2:** Reduced data transmission speeds can have different effects on the performance of an underwater wireless sensor network, depending on the specific communication protocol used, such as Telnet and Superframe. In the case of Telnet, which is a relatively slow protocol, reduced data transmission speeds can result in longer transmission times and higher latency. This can be a concern in applications where real-time data are critical, such as underwater monitoring or surveillance. Longer transmission times can also increase the risk of data loss due to fading or interference, which can further reduce the reliability of the network. In contrast, Superframe is designed to operate at higher data transmission rates, which can result in lower latency and faster data transfer. However, Superframe’s higher data transmission rates can consume more power, which can be a concern in low-power UWSN devices.

Reduced data transmission speeds can also affect the overall network performance of the UWSN. In general, slower data transmission speeds can limit the amount of data that can be transmitted, which can impact the accuracy and timeliness of the data collected by the network. This can have implications for applications that require real-time monitoring or decision making, such as environmental monitoring or underwater exploration.

**Question 3: How do Telnet and Superframe influence the dependability of the UWSN in comparison to other protocols?**

**Answer 3:** Telnet and Superframe can influence the dependability of an underwater wireless sensor network in different ways compared to other protocols. The dependability of a UWSN refers to its ability to maintain reliable and consistent communication among its nodes, even in harsh underwater environments. Telnet is a simple protocol that does not provide any mechanisms for the reliability or error correction. Therefore, its impact on the dependability of a UWSN is limited, and it may not be suitable for applications that require high levels of reliability or data accuracy.

In contrast, Superframe is designed to optimize the dependability of low-power wireless sensor networks, including UWSNs. Superframe incorporates mechanisms such as channel hopping, adaptive modulation, and duty cycling to enhance the reliability of the network. These mechanisms help to reduce interference and fading, minimize energy consumption, and increase network coverage. Superframe can also incorporate mechanisms such as error correction coding to further enhance reliability. When compared to other protocols, the impact of Telnet and Superframe on the dependability of a UWSN depends on the specific application requirements and the environmental conditions. For example, other protocols, such as acoustic communication protocols, may be more reliable in situations where the UWSN needs to operate in highly scattering and absorbing underwater environments. On the other hand, protocols such as Superframe may be more reliable in situations where the UWSN needs to operate in low-power, energy-constrained scenarios.

## 7. Conclusions

Many activities, such as parameter evaluation, monitoring of undersea resources, and military operations planning, occur concurrently while investigating an underwater environment. Because the UWSN can only perform a limited number of tasks, the network’s focus is on the capacity of the network’s batteries. A number of routing protocols, including AODV, LAR1, OLSR, Fisheye, and STAR-LORA, are employed in this study in UWSN networks with a variety of deployment applications, including Telnet and Superframe. These protocols’ performance is examined and contrasted. One of the many statistics measured was the amount of energy utilized during transmit, idle, and receive modes. During the receive mode in the Telnet deployment application, the STAR-LORA routing protocol consumed 0.1 mWh of energy, whereas the LAR1 routing protocol consumed 0.021 mWh of energy in the Superframe deployment application. LAR1 used 0.009 mWh of energy in transmit mode when running the Superframe deployment application, whereas STAR-LORA used 0.05 mWh of energy while running the Telnet deployment application. Both of these measurements were taken using a relatively comparable manner. The application Superframe outperforms the Telnet software in terms of performance. We have limited the study to only energy parameters, and in the future, we will extend our research to other parameters, such as the transmission delay, Percentage of Utilization, End to End delay, BER, throughput, etc.

## Figures and Tables

**Table 1:** CB-UWSN mock-up parameters.

Parameter	Values
Type of Network	CB-UWSN
Channel Model	Underwater Channel
Area (Sq-m)	1500 by 1500 Sq-m
Frequency of the Channel (in Hz)	240 MHz
Number of Nodes	250 nodes
Range (Tx/Rx)	0.05 km
Protocols	AODV, Fisheye, LAR1, OLSR, STAR-LORA
Application	Telnet, Superframe
Time (in s)	500 s
Medium Access Control Protocol	Wireless LANs
Supply Voltage (in volts)	6.5 volts
Packet Size in Words	25
Communication Link	Wireless
Wireless Channel Frequency (in Hz)	1 MHz

**Table 2:** AODV routing protocol parameters investigation using Telnet and Superframe.

Parameter	AODV	
	Telnet	Superframe
Transmit Energy Consumption (mWh)	0.2	0.01
Receive Energy Consumption (mWh)	0.18	0.025
Idle Energy Consumption (mWh)	0.13	0.9

**Table 3:** Fisheye routing protocol parameters investigation using Telnet and Superframe.

Parameter	Fisheye	
	Telnet	Superframe
Transmit Energy Consumption (mWh)	0.8	0.25
Receive Energy Consumption (mWh)	1.2	0.9
Idle Energy Consumption (mWh)	0.16	0.45

**Table 4:** STAR-LORA routing protocol parameters investigation using Telnet and Superframe.

Parameter	STAR-LORA	
	Telnet	Superframe
Transmit Energy Consumption (mWh)	0.05	0.08
Receive Energy Consumption (mWh)	0.1	0.3
Idle Energy Consumption (mWh)	0.15	0.85

**Table 5:** LAR1 routing protocol parameters investigation using Telnet and Superframe.

Parameter	LAR1	
	Telnet	Superframe
Transmit Energy Consumption (mWh)	0.12	0.009
Receive Energy Consumption (mWh)	0.12	0.021
Idle Energy Consumption (mWh)	0.13	0.95

**Table 6:** OLSR routing protocol parameters investigation using Telnet and Superframe.

Parameter	OLSR	
	Telnet	Superframe
Transmit Energy Consumption (mWh)	0.1	0.16
Receive Energy Consumption (mWh)	0.23	0.4
Idle Energy Consumption (mWh)	0.11	0.8

**Table 7:** Energy comparison of all routing protocols.

Protocol	Energy Consumed (mWh) in Receive Mode		Energy Consumed (mWh) in Idle Mode		Energy Consumed (mWh) in Transmit Mode	
	Telnet	Superframe	Telnet	Super Frame	Telnet	Superframe
**AODV**	0.18	0.025	0.13	0.9	0.2	0.01
**Fisheye**	1.2	0.9	0.16	**0.45**	0.8	0.25
**STAR-LORA**	**0.1**	0.3	0.15	0.85	**0.05**	0.08
**LAR1**	0.12	**0.021**	0.13	0.95	0.12	**0.009**
**OLSR**	0.23	0.4	**0.11**	0.8	0.1	0.16

## Data Availability

The datasets used during the current study are available from the corresponding author on reasonable request.

## Abbreviations

Underwater Wireless Sensor Network	UWSN
Cluster-based Underwater Wireless Sensor Network	CB-UWSN
Ad hoc On-demand Distance Vector	AODV
Fisheye State Routing	FSR
Location-Aided Routing 1	LAR1
Optimized Link State Routing Protocol	OLSR
Source Tree Adaptive Routing—Least Overhead Routing Approach	STAR-LORA
Global Positioning System	GPS
Internet of Underwater Things	IoUT
Autonomous Underwater Vehicle	AUV
Base Station	BS
Cluster Head	CH
Sensor Node	SN
Surface Station	SS
Multi-Point Relay	MPR
Telecommunication Network	Telnet
Superframe	S-frame
Transmission Control Protocol	TCP
Contention Access Period	CAP
Contention Free Period	CFP
Guaranteed Time Slot	GTS

## References

[1] Srinivasulu A., Anand Kumar G. (2022). Performance Analysis of Underwater Wireless Sensor Network by Deploying FTP, CBR, and VBR as Applications. J. Comput. Netw. Commun..

[2] Luo J., Fan L., Wu S., Yan X. (2018). Research on localization algorithms based on acoustic communication for underwater sensor networks. Sensors.

[3] Heidemann J., Ye W., Wills J., Syed A., Li Y. Research challenges and applications for underwater sensor networking. Proceedings of the Wireless Communications and Networking Conference (WCNC 2006).

[4] Yang J., Fei Z., Shen J. (2015). Hole detection and shape-free representation and double landmarks based geographic routing in wireless sensor networks. Digit. Commun. Netw..

[5] Sathish K., Ravikumar C.V., Srinivasulu A., Rajesh A., Oyerinde O.O. (2022). Performance and Improvement Analysis of the Underwater WSN Using a Diverse Routing Protocol Approach. J. Comput. Netw. Commun..

[6] Anbazhagan R., Venkata R.C., Arena F., Pau G. (2022). Investigation and Numerical Simulation of the Acoustic Target Strength of the Underwater Submarine Vehicle. Inventions.

[7] Venkata R.C., Anbazhagan R., Pau G. (2023). Review of Localization and Clustering in USV and AUV for Underwater Wireless Sensor Networks. Telecom.

[8] Pughat A., Sharma V. (2017). Performance analysis of an improved dynamic power management model in wireless sensor node. Digit. Commun. Netw..

[9] Alkindi Z., Alzeidi N., Touzene B.A.A. (2018). Performance evolution of grid based routing protocol for underwater wireless sensor networks under different mobile models. Int. J. Wirel. Mob. Netw..

[10] Yildiz H.U., Gungor V.C., Tavli B. (2018). Packet size optimization for lifetime maximization in underwater acoustic sensor networks. IEEE Trans. Ind. Inform..

[11] Bhattacharya K., Alam S., De D. (2019). CUWSN: Energy efficient routing protocol selection for cluster-based underwater wireless sensor network. Microsyst. Technol..

[12] Wang K., Gao H., Xu X., Jiang J., Yue D. (2016). An energy-efficient reliable data transmission scheme for complex environmental monitoring in underwater acoustic sensor networks. IEEE Sens. J..

[13] Mohan P., Subramani N., Alotaibi Y., Alghamdi S., Khalaf O.I., Ulaganathan S. (2022). Improved Metaheuristics-Based Clustering with Multihop Routing Protocol for Underwater Wireless Sensor Networks. Sensors.

[14] Zandi R., Kamarei M., Amiri H. Underwater acoustic sensor network localization using four directional beams. Proceedings of the 2013 21st Iranian Conference on Electrical Engineering (ICEE).

[15] Cui J.H., Kong J., Gerla M., Zhou S. (2006). The challenges of building mobile underwater wireless networks for aquatic applications. IEEE Netw..

[16] Sathish K., Hamdi M., Chinthaginjala R., Pau G., Ksibi A., Anbazhagan R., Abbas M., Usman M. (2023). Reliable Data Transmission in Underwater Wireless Sensor Networks Using a Cluster-Based Routing Protocol Endorsed by Member Nodes. Electronics.

[17] Li J., Gao H., Zhang S., Chang S., Chen J., Liu Z. (2016). Self-localization of autonomous underwater vehicles with accurate sound travel time solution. Comput. Electr. Eng..

[18] Kalapraveen B. (2017). Receiver design using artificial neural network for signal detection in MC-CDMA system. Int. J. Intell. Eng. Syst..

[19] Mridula K.M., Ameer P.M. (2018). Localization under anchor node uncertainty for underwater acoustic sensor networks. Int. J. Commun. Syst..

[20] Han G., Jiang J., Shu L., Xu Y., Wang F. (2012). Localization algorithms of underwater wireless sensor networks: A survey. Sensors.

[21] Moore R.K. (1963). Effects of a Surrounding Conducting Medium on Antenna Analysis. IEEE Trans. Antennas Propag..

[22] Wait J.R., Collin R.E., Zucker F.J. (1969). Electromagnetic Fields of Sources in Lossy Media. Antenna Theory.

[23] Bagadi K., Ravikumar C.V., Sathish K., Alibakhshikenari M., Virdee B.S., Kouhalvandi L., Olan-Nuñez K.N., Pau G., See C.H., Dayoub I. (2022). Detection of Signals in MC–CDMA Using a Novel Iterative Block Decision Feedback Equalizer. IEEE Access.

[24] Agarwal R., Kumar S., Hegde R.M. (2015). Algorithms for crowd surveillance using passive acoustic sensors over a multimodal sensor network. IEEE Sens. J..

[25] Sathish K. (2022). Underwater Wireless Sensor Network Performance Analysis Using Diverse Routing Protocols. J. Sens. Actuator Netw..

[26] Alsulami M., Elfouly R., Ammar R. A reliable underwater computing system. Proceedings of the 2021 4th IEEE International Conference on Industrial Cyber-Physical Systems (ICPS).

[27] Erol M., Vieira L.F., Caruso A., Paparella F., Gerla M., Oktug S. Multi stage underwater sensor localization using mobile beacons. Proceedings of the 2008 Second International Conference on Sensor Technologies and Applications (sensorcomm 2008).

[28] Teja G.S., Samundiswary P. Performance analysis of DYMO protocol for IEEE 802.15. 4 based WSNs with mobile nodes. Proceedings of the 2014 International Conference on Computer Communication and Informatics.

[29] Patil M.S.A., Mishra M.P. (2017). Improved mobicast routing protocol to minimize energy consumption for underwater wireless sensor networks. Int. J. Res. Sci. Eng..

[30] Manjula S.H., Abhilash C.N., Shaila K., Venugopal K.R., Patnaik L.M. (2008). Performance of AODV routing protocol using group and entity mobility models in wireless sensor networks. Int. Multi Conf. Eng. Comput. Sci..

[31] Han L., Li Z., Liu W., Qu W., Nie L., Zheng L., Liu M. Sensor localization in underwater sensor networks using distance transform based skeleton extraction. Proceedings of the 2016 2nd IEEE International Conference on Computer and Communications (ICCC).

[32] Subramani N., Mohan P., Alotaibi Y., Alghamdi S., Khalaf O.I. (2022). An Efficient Metaheuristic-Based Clustering with Routing Protocol for Underwater Wireless Sensor Networks. Sensors.

[33] Hou R., Fu J., Dong M., Ota K., Zeng D. (2022). An Unequal Clustering Method Based on Particle Swarm Optimization in Underwater Acoustic Sensor Networks. IEEE Internet Things J..

[34] Xing G., Chen Y., Hou R., Dong M., Zeng D., Luo J., Ma M. (2021). Game-Theory-Based Clustering Scheme for Energy Balancing in Underwater Acoustic Sensor Networks. IEEE Internet Things J..

[35] Tian W., Zhao Y., Hou R., Dong M., Ota K., Zeng D., Zhang J. (2023). A Centralized Control-Based Clustering Scheme for Energy Efficiency in Underwater Acoustic Sensor Networks. IEEE Trans. Green Commun. Netw..

